# Molecular profiling of IgE sensitization to dermatophagoides allergen components: clinical correlates in a high-exposure region

**DOI:** 10.3389/falgy.2026.1816355

**Published:** 2026-06-12

**Authors:** David El-Qutob, María Dolores Martos Calahorro, Ángel Ferrer Torres, Pilar Alba Jordá, Ramón López Salgueiro, Luis Ángel Navarro Seisdedos

**Affiliations:** 1 Allergy Department, University Hospital of La Plana, Vila-real, Spain; 2 Allergy Department, Hospital Universitario de Torrevieja, Torrevieja, Spain; 3 Allergy Department, Hospital de la Vega Baja, Orihuela, Spain; 4 Allergy Department, Hospital de Manises, Manises, Spain; 5 Allergy Department, Hospital Universitari I Politecnic La Fe, Valencia, Spain; 6 Allergy Department, Hospital Lluis Alcanyis de Xativa, Xàtiva, Spain

**Keywords:** alergens, allergen, dermatophagoides, component-resolved diagnosis, house dust mite, specific IgE

## Abstract

**Introduction:**

Respiratory allergies, mediated by IgE reactions to house dust mites (HDM), constitute a significant disease burden in Mediterranean climates. We investigated molecular sensitization profiles to *Dermatophagoides* allergens and their clinical correlations in a high-exposure region.

**Material and methods:**

This prospective multicentre study enrolled patients (5–65 years) with HDM-allergic rhinitis/asthma from the Valencian Community, Spain. Sensitization to Dermatophagoides components (Der *p* and Der f) was analysed using ALEX^2^ microarray (positivity boundary IgE ≥0.30 kUA/L). Statistical analyses included descriptive statistics, correlation tests, and multivariate regression.

**Results:**

Der *p* 2 (92.7%), Der f 2 (92.7%), Der *p* 1 (87%), Der p 23 (83.7%), Der f 1 (82.9%), and Der p 7 (51.2%) were the most prevalent sensitization types among 118 participants (median age 29; 53% female). Overall, 36 sensitization profiles emerged, half of which were grouped into 4 dominant profiles: profile 1 (14.4%), profile 5 (13.6%), profile 11 (6.8%), and profile 2 (95.1%), all characterized by core allergens Der p 1, Der f 1, Der p 2, and Der f 2, with varying additional components (e.g., Der p 5, 7, 21, 23). Younger age predicted higher Der p 23 reactivity (*p* < 0.05), while females showed lower Der p 7/23 sensitization. Poorly controlled rhinitis (93% of cases) and asthma correlated with Der p 1/Der f 1 responses.

**Conclusion:**

The study reveals diverse HDM sensitization profiles in a Mediterranean population, highlighting the clinical relevance of both major and minor allergens. Component-resolved diagnostics may support a better understanding of the heterogeneity of HDM sensitization profiles in high-exposure regions.

## Introduction

Respiratory allergy refers to a hypersensitive reaction of the immune system to airborne allergens, resulting in conditions such as allergic rhinitis and allergic asthma (1). These reactions are mediated primarily by immunoglobulin E (IgE), which triggers inflammatory responses upon exposure to specific allergens (1). Various factors—including age, climate, ethnicity, genetic predisposition, environmental exposure, lifestyle habits, and socioeconomic status—can influence the prevalence, severity, and sensitization patterns associated with these allergic conditions (1). For instance, allergen sensitization to house dust mites is predominant in coastal areas, while pollen sensitization is more prevalent in other areas (2).

The prevalence of respiratory allergy has increased significantly worldwide over recent decades (3). Globally, it is estimated that up to 30% of the population may be affected by some form of allergic rhinitis (4). In Europe, this figure is slightly higher, with approximately 25%–40% of individuals suffering from allergic respiratory conditions (5). Spain exhibits similar prevalence rates (5), and within the Valencian Community, the numbers reflect the national trend, indicating that a substantial proportion of the population experience allergic symptoms (6).

Respiratory allergies—including rhinitis, rhinoconjunctivitis, and asthma—impose a considerable burden on multiple levels. From a health perspective, they are associated with reduced lung function, poor sleep quality, and increased susceptibility to other respiratory infections (7). In terms of quality of life, affected individuals often report limitations in daily activities, impaired cognitive performance, and emotional distress (8). Socially and economically, respiratory allergies lead to reduced work productivity, increased absenteeism, and a significant demand on healthcare systems due to chronic treatment and recurrent medical consultations (8). To put this into context, rhinitis is the primary reason for seeking specialist care for allergic disorders in Spain, accounting for 54.1% of visits.

Among the various allergens responsible for respiratory allergies, house dust mites (HDM) are particularly significant (9). These microscopic arthropods are commonly found in indoor environments and thrive in warm and humid conditions. In Spain, the most relevant mite species are those of the *Dermatophagoides* genus, including *D. pteronyssinus* (DP) and *D. farina* (DF) (10, 11). The prevalence and type of mite species vary according to regional climate. In the Mediterranean coast, particularly the city of Valencia, the high humidity and moderate temperatures create ideal conditions for the proliferation of DP, making it a primary source of allergic sensitization in this area (10).

*Dermatophagoides* mites contain a complex mixture of allergenic components. According to molecular diagnostic platforms such as ALEX^2^ (Allergy Explorer), these include major allergens such as *Der p 1* and *Der p 2*, along with components traditionally considered minor—such as *Der p 5, Der p 7, Der p 10, and Der p 21*—though their frequent detection suggests they may play a significant role (12–16). Each of these components can provoke specific IgE (sIgE) responses, helping to define the patient's sensitization profile.

Understanding the immunofrequency of these components within specific populations is essential for improving treatment strategies, as allergen-specific immunotherapy relies on accurate identification of relevant sensitization patterns (12). Literature supports the existence of patient subgroups with distinct sensitization profiles, suggesting that treatment approaches should be tailored accordingly (12). Studies have demonstrated that patients sensitized only to major allergens may respond differently to immunotherapy than those with broader sensitization patterns involving minor allergens (13).

The aim of this study is to analyse the recognition profiles of sIgE to allergenic components of *Dermatophagoides* (DP/DF) in sensitized individuals residing in a high-exposure area. The study also explores the relationship between these sensitization profiles and clinical variables, in order to contribute to a better understanding of HDM sensitization and advance the development of more effective allergy management strategies.

## Material and methods

### Design and population

This was an observational multicentre study conducted between 2021 and 2022 in 10 hospitals in the Valencian Community, Spain.

The study population included individuals between the ages of 5 and 65 years who had been diagnosed with allergic rhinitis, with or without asthma, due to hypersensitivity to DP and/or DF. Eligible participants had experienced symptoms for at least 1 year prior to enrolment and were considered candidates for DP/DF allergen immunotherapy. Sensitization to Dermatophagoides was confirmed at inclusion using both skin prick tests (SPT) and specific IgE (ImmunoCAP), as required by the inclusion criteria (SPT ≥ 3 mm and sIgE ≥ 0.7 kU/L). Patients were excluded if they had clinically relevant sensitization to other allergens, had received specific immunotherapy containing *Dermatophagoides* within the previous 5 years, or were undergoing treatment with anti-IgE antibodies (such as omalizumab) or other biological therapies that could potentially influence clinical or analytical outcomes. Sensitization to storage mites (e.g., *Lepidoglyphus destructor*) or *Blomia tropicalis* was not treated as a specific standalone exclusion criterion. Instead, patients were only excluded if the investigator deemed the sensitization to these other mites to be the main contributor to their clinical symptoms. Clinical relevance is established, once sensitization to an allergen has been identified ither through ALEX or specific IgE (ImmunoCAP), by correlating the patient's clinical symptoms with the detected sensitization.

A total of 124 patients with *Dermatophagoides*-allergic rhinitis and/or asthma were initially enrolled. After assessment of eligibility criteria and data completeness, 6 patients were excluded from the final analysis due to having received previously HDM-specific immunotherapy. Consequently, the final study population consisted of 118 patients.

The study protocol received approval from the independent ethics committee of Hospital Arnau-Vilanova in July 2021. All procedures followed the ethical standards outlined in the Declaration of Helsinki and complied with the EU General Data Protection Regulation (GDPR). Personal identifiers were fully anonymized in all reported data.

### Variables

The recorded variables included sex, age, medical and surgical history, concomitant medication use, asthma and/or rhinitis diagnosis, and the degree of asthma/rhinitis control. Concomitant medication refers to any treatment resulting from common conditions at the time of evaluation (as hypertension, diabetes, dyslipidaemia, hypothyroidism, psychiatric disorder, etc.). This information was obtained from medical records and registered in an *ad-hoc* eCRF.

The classification of asthma severity and degree of control according to the Spanish guidelines for the management of asthma was used (14). The severity and control of rhinitis was classified according to the criteria of the American Academy of Allergy, Asthma & Immunology (AAAAI) and the American College of Allergy, Asthma and Immunology (ACAAI) guidelines (15).

IgE measurements specific to DP and DF were carried out in accordance with standard clinical practice ImmunoCAP (Thermo Fisher) in order to assess the patient's degree of sensitisation to the allergens suspected of causing clinical symptoms.

sIgE was measured using the ALEX^2^ microarray, a multiplex test that includes 295 allergens, comprising 117 whole allergen extracts and 178 molecular components (17). In this study, the analysis was specifically directed at sensitization to allergenic components of *Dermatophagoides* (ALEX^2^ results were provided after the clinical evaluation). The sIgE levels were measured for DP components *Der p 1, Der p 2, Der p 5, Der p 7, Der p 10, Der p 11, Der p 20, Der p 21, and Der p 23,* as well as for DF components *Der f 1 and Der f 2*. These tests were carried out according to routine practice at each centre. A sensitization response was considered positive when sIgE levels were equal to or greater than 0.30 kUA/L.

### Ethics

The study was reviewed and approved by the Research Ethics Committee with Medicinal Products (CEIm) of Hospital Arnau de Vilanova-Llíria, Valencia, Spain, which issued a favourable opinion on 28 July 2021 (meeting minutes No. 9/2021). The study was also registered in the Spanish Agency of Medicines and Medical Devices (AEMPS) GESTO platform for observational studies under code 0045-2021-OBS. All procedures were conducted in accordance with the Declaration of Helsinki and applicable data protection regulations.

### Statistical analysis

A descriptive analysis was performed using median and interquartile range (IQR) for continuous variables, and absolute and percentage frequencies for categorical variables.

Normality was assessed using the Shapiro–Wilk test. Inferential analyses included the Mann–Whitney U test, the Kruskal–Wallis test, and correlation analysis by Spearman's rank correlation measure. As a proxy assessment of sensitization due to exposure duration, a correlation between age and IgE levels was estimated.

For the multivariate analysis, binary logistic regression models were employed to explore the association between molecular sensitization and clinical/demographic variables. To mitigate the risk of overfitting, a two-step variable selection procedure was implemented: (i) a bivariate analysis was initially conducted to screen potential predictors, including only those with *p* < 0.200 in the initial multivariate model; and (ii) a backward stepwise selection method was subsequently used to achieve the most parsimonious model. Results are expressed as Odds Ratios (OR) with their corresponding 95% Confidence Intervals (CI) and *p*-values.

All statistical analyses were performed using R version 4.2.0.

## Results

### Demographics and clinical characteristics

A total of 118 patients were included in the study with a median age of 29 years (IQR 18–37; Table 1). Of these, 62 patients, representing 53% of the cohort, were female; 13% of the participants reported relevant medical history related to asthma/rhinitis diagnosis (for example, allergic conjunctivitis, atopic dermatitis, food allergy); and 12% were receiving concomitant medication at the time of evaluation. Asthma was present in 35% of patients, with a predominance of mild persistent asthma. Among those with asthma, 46% had mild persistent symptoms, while 22% were classified as having moderate persistent asthma. Rhinitis was highly prevalent, affecting 97% of patients. Among those with rhinitis, 89% experienced symptoms of moderate intensity, and a striking 93% had rhinitis that was either uncontrolled or only partially controlled. The complete demographics are shown in Table 1.

**Table 1:** Demographics and clinical characteristics (*n*=118).

Age (years), median (IQR)	29 years (18–37)
Female patients, *n* (%)	62 (53)
Atopic and other comorbidities^a^, *n* (%)	62 (53)
Asthma (any)	
• Size group, n	41 (35)
• Severity, *n* (%)^b^	
Intermittent	13 (21)
Mild persistent	19 (46)
Moderate/severe persistent	9 (22)
• Control, *n* (%)^b^	
Controlled	16 (41)
Partially controlled	18 (46)
Uncontrolled	5 (13)
Rhinitis (any)	
• Size group, n	115 (97)
• Intensity, *n* (%)^b^	
Mild	9 (8)
Moderate	76 (68)
Severe	27 (24)
• Control, *n* (%)^b^	
Controlled	8 (7)
Partially controlled	68 (60)
Uncontrolled	37 (33)

a Include allergic conjunctivitis, atopic dermatitis, and food allergies..

b The proportion was estimated based on the respective asthma or rhinitis patient groups with available data.

Regarding sensitization to aeroallergens, all patients were sensitized to DP, while 97% showed sensitization to DF, and 22% were positive for *Lepidoglyphus destructor* sensitization (Table 2). Additionally, 68% and 21% of the cohort were sensitized to pollens and animal dander, respectively.

**Table 2:** Sensitization to aeroallergens.

Aeroallergen	Sensitization, *n* (%)
*Dermatophagoides pteronyssinus*	118 (100)
*Dermatophagoides farinae*	114 (97)
*Lepidoglyphus destructor*	26 (22)
Pollens	80 (68)
Animal dander	25 (21)

### IgE sensitization profiles

**Figure 1:**
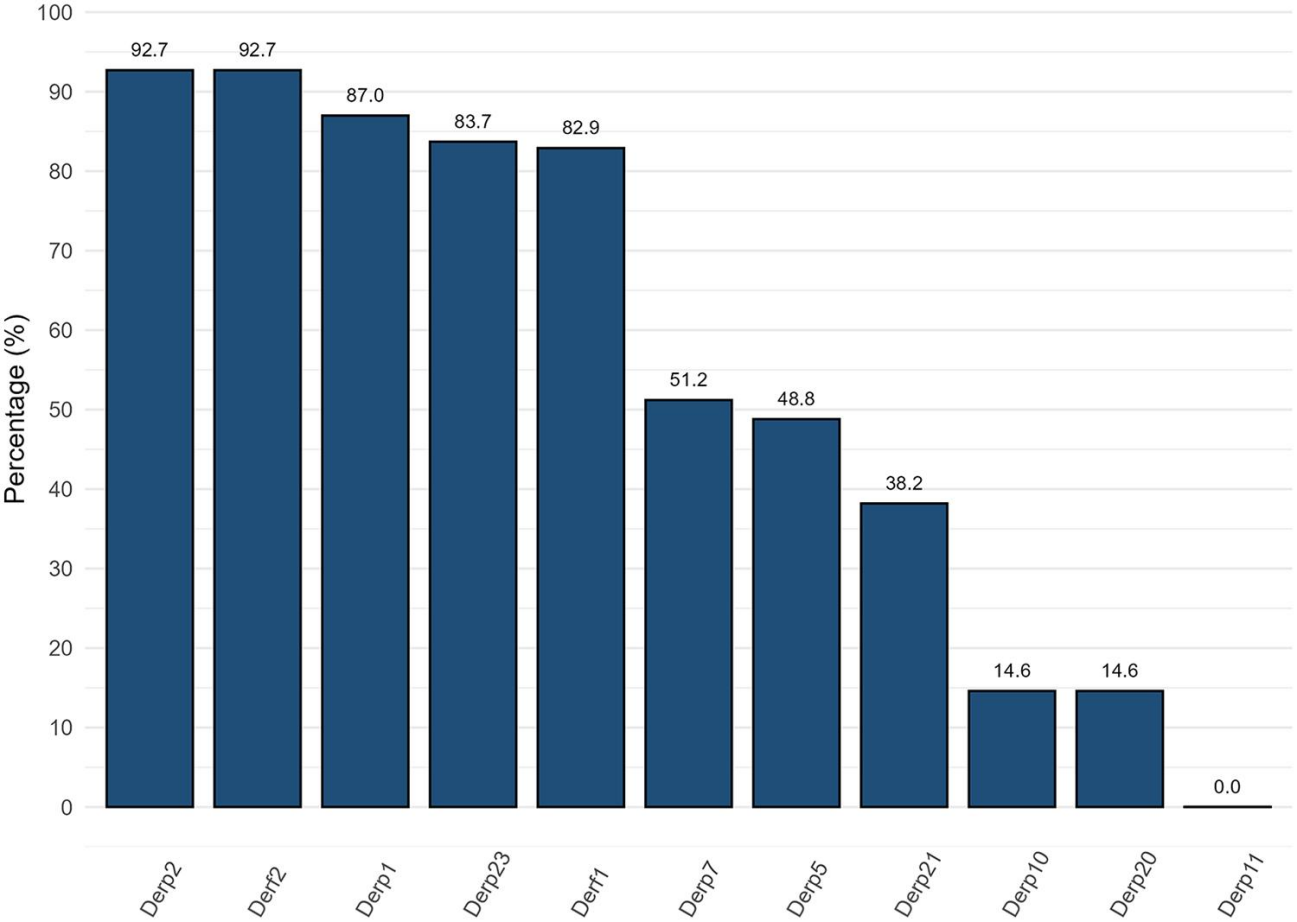
Prevalence of IgE sensitization to specific molecular components of D. pteronyssinus and D. farinae (positivity cut-off point ≥0.30 kUA/L).

The ALEX^2^ macroarray identified positive sensitization to multiple molecular components of *Dermatophagoides* (Figure 1). The most frequently recognized components were *Der p 2* (92.7%), *Der f 2* (92.7%), *Der p 1* (87%), *Der p 23* (83.7%), and *Der f 1* (82.9%).

The study identified 36 distinct sensitization profiles based on IgE response patterns. Overall, the distribution of profiles indicates a high degree of heterogeneity in individual sensitization patterns. While major allergens such as Der p 1, Der f 1, Der p 2, and Der f 2 were consistently present across many profiles, the inclusion of mid- and minor-tier components (e.g., Der p 5, Der p 7, Der p 10, Der p 21) revealed complex and variable IgE response profiles within the population. Half of the participants could be grouped into 4 main profiles, each characterized by specific combinations of *Dermatophagoides* components. Profile 1, the most prevalent, included individuals sensitized to *Der p 1, Der f 1, Der p 2, Der f 2, and Der p 23* and accounted for 14.4% of the total cohort. Profile 5, representing 13.6%, was nearly as common and was defined by reactivity to a broader range of components including *Der p 5, Der p 7, Der p 21, and Der p 23*, in addition to the major allergens *Der p 1, Der f 1, Der p 2, and Der f 2.* Other profiles were also significant.

Profile 11 accounted for 6.8% and includes four core allergens, as well as sensitisation to *Der p 7* and *Der p 23*. By contrast, Profile 2 (5.1% of the sample) was characterised by a sensitisation pattern that was restricted to these four core allergens, with no reactivity to other analysed components. The composition of the profiles with a prevalence of over 2% in the sample is detailed in Table 3.

**Table 3:** ALEX² sensitization profiles observed in more than 2% of patients.

Profile	Components	N	Percentage
1	Der p 1, Der f 1, Der p 2, Der f 2, Der p 23	17	14.4
5	Der p 1, Der f 1, Der p 2, Der f 2, Der p 5, Der p 7, Der p 21, Der p 23	16	13.6
11	Der p 1, Der f 1, Der p 2, Der f 2, Der p 7, Der p 23	8	6.8
2	Der p 1, Der f 1, Der p 2, Der f 2	6	5.1
7	Der p 1, Der f 1, Der p 2, Der f 2, Der p 5, Der p 7, Der p 23	6	5.1
9	Der p 2, Der f 2	6	5.1
19	Der p 2, Der f 2, Der p 23	5	4.2
13	Der p 1, Der f 1, Der p 2, Der f 2, Der p 5, Der p 21, Der p 23	4	3.4
20	Der p 1, Der f 1	4	3.4
10	Der p 1, Der f 1, Der p 23	3	2.5
12	Der p 1, Der f 1, Der p 2, Der f 2, Der p 7, Der p 10, Der p 23	3	2.5

### IgE correlation analysis

**Figure 2:**
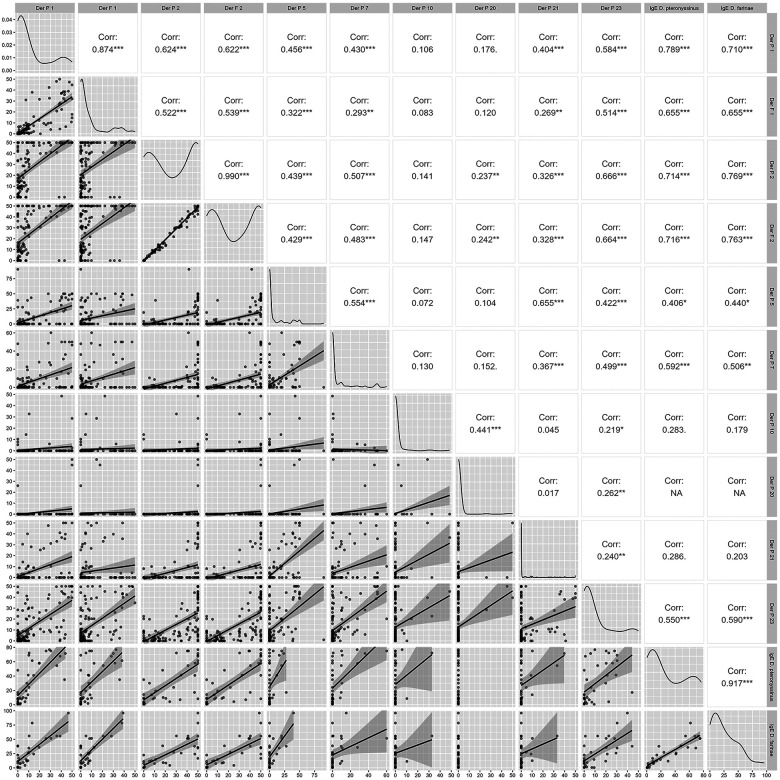
Correlation matrix between ALEX² component results and IgE results for D. pteronyssinus and D. farinae. The upper-right triangular portion presents Spearman correlation coefficients. Statistical significance is indicated by asterisks: * *p* < 0.05, ** *p* < 0.01, and *** *p* < 0.001. The lower-left triangular portion shows scatterplots illustrating the distribution of values between each pair of variables. The diagonal panels display the density plots for each variable.

Figure 2 displays the correlation matrix assessing the relationships between sIgE levels obtained through the ALEX^2^ platform for individual *Dermatophagoides* components and total sIgE levels for DP and DF identified by ImmunoCAP. Strong positive correlations were observed between several ALEX^2^ components and the total IgE for DP and DF. Notably, *Der p 1, Der f 1, Der p 2* and *Der f 2* exhibited very high correlation coefficients (r > 0.85) with total IgE, highlighting their major allergenic relevance. For instance, *Der p 2* and *Der f 2* showed an almost perfect correlation (r = 0.98), suggesting considerable overlap in patient sensitization profiles.

Moderate correlations were found between *Der p 5, Der p 7*, and *Der p 21* with total sIgE, whereas weaker correlations (r < 0.5) were observed for components such as *Der p 10* and *Der p 20*. Statistically significant correlations are marked with asterisks, indicating the strength and consistency of the observed relationships.

Age showed a weak-to-moderate inverse correlation with IgE to DP (r = −0.44, *p* = 0.006) and a non-significant inverse correlation with IgE to DF (r = −0.33, *p* = 0.071). IgE levels to DP and DF were strongly correlated with each other (0.92, *p* < 0.001).

### Factors related to *dermatophagoides* sensitization

Table 4 summarizes the multivariate analysis of sIgE in relation to demographics and clinical characteristics. A strong positive association was observed between *Der p 5* sensitization and female gender (OR = 8.47, *p* = 0.003). By comparison, *Der p 23, Der p 7,* and *Der p 21* showed inverse associations with female gender. Of these, *Der p 23* reached statistical significance (OR = 0.28, *p* = 0.018), while *Der p 7* and *Der p 21* showed marginal significance (p≈0.05–0.07). Regarding age, sensitization to *Der f 1* was significantly associated with younger participants (OR = 3.40, *p* = 0.005), while *Der p 23* showed a non-significant trend (*p* = 0.059). *Der p 1* sensitization showed a non-significant positive association with concomitant medication use (OR = 4.84, *p* = 0.066), while *Der p 23* (OR = 0.27, *p* = 0.048) and *Der p 7* (*p* = 0.057) were inversely associated.

**Table 4:** Multivariate analysis of sIgE to dermatophagoides molecular components with demographics and clinical characteristics.

Component	Outcome	OR	*p*-value	R^2^ Tjur
Der p 5	**Female sex**	**8.47**	**0.003**	0.177
Der p 23	**Female sex**	**0.28**	**0.018**	0.177
Der p 7	Female sex	0.34	0.051	0.177
Der p 21	Female sex	0.31	0.073	0.177
Der f 1	**Age <30**	**3.44**	**0.005**	0.118
Der p 23	Age <30	2.45	0.059	0.118
Der p 1	Concomitant medication	4.84	0.066	0.137
Der p 23	**Concomitant medication**	**0.27**	**0.048**	0.137
Der p 7	Concomitant medication	0.12	0.057	0.137
Der p 1	**Persistent rhinitis**	**4.02**	**0.028**	0.048
Der f 1	**Uncontrolled rhinitis**	**3.12**	**0.025**	0.076
Der f 1	Persistent asthma	**9.7**	0.056	0.178
Der p 1	**Persistent asthma**	**0.03**	**0.028**	0.178
Der f 1	**Uncontrolled asthma**	**0.04**	**0.033**	0.153
Der p 1	**Uncontrolled asthma**	**0.04**	**0.033**	0.153
Der f 1	Uncontrolled asthma	**5.67**	0.14	0.153

Odds ratios (OR) were obtained from multivariable logistic regression models assessing the association between sensitization to individual molecular components and demographic or clinical characteristics. OR >1 indicate a positive association with the outcome, whereas OR <1 indicate an inverse association. Statistically significant associations were defined as *p* < 0.05 and are highlighted in bold and green. Trends were defined as *p* values between 0.05 and 0.10 and are highlighted in yellow. Tjur's R² is shown as a measure of model discrimination.

In terms of clinical outcomes, *Der p 1* sensitization was significantly associated with persistent rhinitis (OR = 4.00, *p* = 0.028). Similarly, *Der f 1* was significantly associated with uncontrolled rhinitis (OR = 3.12, *p* = 0.025). *Der f 1* also had a strong but non-significant association with persistent asthma (OR = 9.70, *p* = 0.056). In contrast, *Der p 1* was significantly inversely associated with both persistent asthma (OR = 0.03, *p* = 0.028) and uncontrolled asthma (OR = 0.04, *p* = 0.033).

### Identification of sIgE sensitization profiles

Table 5 presents the multivariate analysis of sIgE profiles in relation to demographics and clinical characteristics. No statistically significant associations were observed between sensitization profiles and gender. However, profile 1 showed a trend towards a higher frequency among males (OR = 0.23, *p* = 0.074), whereas profile 2 was more common among females (OR = 3.23, *p* = 0.164), albeit with wide confidence intervals.

**Table 5:** Multivariate analysis of IgE profiles with demographics and clinical characteristics.

Profile	Outcome	OR	*p*-value	R^2^ Tjur
Profile 1	Female sex	0.23	0.074	0.032
Profile 1	Persistent rhinitis	3.82	0.054	0.037
Profile 1	Uncontrolled rhinitis	0.26	0.085	0.068
Profile 1	Concomitant medication	0.12	0.010	0.079
Profile 2	Female sex	3.23	0.164	0.032
Profile 2	**Age <30**	**3.61**	**0.001**	0.092
Profile 2	Severe rhinitis	2.25	0.099	0.025
Profile 2	**Uncontrolled rhinitis**	**4.51**	**0.042**	0.068
Profile 2	**Concomitant medication**	**5.33**	**0.037**	0.079
Profile 5	Uncontrolled rhinitis	0.33	0.141	0.068
Profile 11	Uncontrolled rhinitis	2.57	0.145	0.068
Profile 11	Asthma diagnosis	1.82	0.145	0.018

Odds ratios (OR) were obtained from multivariable logistic regression models assessing the association between sensitization to individual molecular components and demographic or clinical characteristics. OR >1 indicate a positive association with the outcome, whereas OR <1 indicate an inverse association. Statistically significant associations were defined as *p* < 0.05 and are highlighted in bold and green. Trends were defined as *p* values between 0.05 and 0.10 and are highlighted in yellow. Tjur's R² is shown as a measure of model discrimination.

A significant association was identified between profile 2 and younger age (<30 years): individuals who exhibited this profile were more than 3 times as likely to be under 30 (OR = 3.61, *p* = 0.001). In the analysis of concomitant medication use, profile 2 was significantly associated with increased medication use (OR = 5.33, *p* = 0.037). In contrast, profile 1 demonstrated a significant inverse association (OR = 0.12, *p* = 0.010).

Profile 11 showed a non-significant trend towards a higher frequency in asthma patients (OR =  1.82, *p* = 0.145). Profile 1 was marginally associated with persistent rhinitis (OR = 3.82, *p* = 0.054), a finding that may warrant further investigation. Similarly, profile 2 exhibited a non-significant trend toward association with more severe rhinitis (OR = 2.25, *p* = 0.099).

Regarding rhinitis control, profile 2 was significantly associated with uncontrolled rhinitis (OR = 4.50, *p* = 0.040). By comparison, profiles 1 and 5 showed inverse but non-significant associations (OR = 0.26 and 0.33, respectively), while profile 11 was not clearly associated with rhinitis (OR = 2.57, *p* = 0.145).

## Discussion

To our knowledge, this study is the first to characterize molecular sensitization patterns to HDM allergens in an adult population in the Valencian Community, Spain. It also provides comprehensive data on demographic and clinical variables associated with these sensitization profiles.

Before comparing our findings with those of other studies, it is important to first highlight key characteristics of our study population. This cohort comprised both paediatric and adult patients, ranging in age from 5 to 65 years. Rhinitis was highly prevalent, affecting 97% of participants; among these, 89% experienced symptoms of moderate intensity and 93% had rhinitis that was either uncontrolled or only partially controlled. Asthma was diagnosed in 35% of patients, the most common form being mild persistent asthma. All participants resided in the Valencian Community, a Mediterranean region in eastern Spain that includes both coastal and inland areas. This region is characterized by warm temperatures and relatively high humidity for most of the year. Valencia is described as Csa according to the Köppen–Geiger classfication (16), with its hot-summer Mediterranean climate, marked by dry summers and mild, wet winters. These climatic conditions—especially prevalent in coastal areas—create an environment that supports the proliferation of *Dermatophagoides* species, making this region particularly relevant for investigating HDM-related allergic diseases.

Given the specific environmental conditions of the Valencian Community and the demographic profile of our cohort, it is useful to contextualize our findings within the broader landscape of molecular sensitization studies in Spain.

Our findings from the Valencian cohort align with broader national data on molecular sensitization to *Dermatophagoides* allergens, while also underscoring notable regional and population-specific variations. In our study, *Der p 2* (92.7%), *Der f 2* (92.7%), *Der p 1* (87%) and *Der p 23* (83.7%) were the most frequently recognized allergens, mirroring trends observed in studies from Tenerife (18, 19) and Barcelona (20), where these major allergens consistently showed high prevalence. These similarities may be attributed to comparable environmental and climatic conditions: Valencia, Barcelona, and Tenerife all share a climate marked by warm temperatures and high relative humidity, which could promote the proliferation of similar HDM species.

Compared to the study by López-Rodríguez et al. conducted in A Coruña, which reported lower sensitization rates to *Der p 1* (66%) and *Der p 5* (44%) (21), our population exhibited a higher prevalence of sensitization to these components—87% and 48.8%, respectively. This disparity suggests that environmental and demographic factors may play a significant role in shaping sensitization profiles. A Coruña, a city in Galicia, Spain, experiences a temperate oceanic climate (Cfb in the Köppen–Geiger classification) (16), characterized by mild winters, warm but not hot summers, and consistent year-round precipitation resulting in high ambient humidity. Such climatic conditions may promote the proliferation of indoor allergens like DP, thereby influencing regional patterns of allergic sensitization. In our study population, the observed sensitization prevalence was 87%, higher than the moderate *Der p 1* IgE (7.4 ± 13.6 kU/L) levels reported in a previous study conducted by Martínez-Cañavate et al. in a paediatric population (22). This discrepancy could reflect an age-related increase in sensitization breadth, as our cohort included both adults and children. Minor allergens such as *Der p 10*, *Der p 20*, and *Der p 21* consistently showed low prevalence in all studies, including ours. The near absence of *Der p 10* sensitization in our sample is consistent with the findings of the publications by Martínez-Cañavate's, López-Rodríguez and Jiménez-Feijoo (21, 23, 24). These differences reinforce the value of regional allergen profiling and highlight how age, environmental exposure, climate (e.g., Mediterranean humidity), and local mite species may influence molecular sensitization patterns.

Overall, our results support the growing consensus that while major HDM allergens remain central to diagnosis, sensitization to minor and mid-tier allergens can vary significantly across populations and should not be overlooked. Inclusion of a broader range of components in diagnostic panels may improve detection and facilitate more tailored therapeutic approaches (25), particularly in areas with unique environmental profiles like the Valencian Mediterranean coast. Together, these comparisons highlight the importance of region-specific allergen profiling and support the inclusion of both major and minor HDM components in molecular diagnostic panels (25). The use of diagnostic panels may improve the identification of more frequent sensitization profiles to improve therapeutic approaches. This strategy not only enhances diagnostic precision but also supports the development of immunotherapy plans, particularly for populations exposed to unique environmental conditions such as those in Mediterranean coastal regions.

The analysis of sensitization profiles revealed that while a substantial proportion of individuals demonstrated strong sIgE reactivity to major components (*Der p 1, Der p2, Der f 1* and *Der f 2*), this pattern was not universal. Approximately half of the participants showed additional sensitization to other components, indicating a more complex immunological profile than can be explained by major allergens alone. Notably, some patients did not exhibit sIgE recognition to the most commonly reactive components, further highlighting the heterogeneous nature of sensitization within this population. A high correlation between the major allergens and total IgE for both *Dermatophagoides* species was observed. Notably, the correlation between *Der p 2* and *Der f 2* was almost perfect (r = 0.98), indicating a massive overlap in sensitization profiles. This exceptionally high interspecies correlation for Group 2 allergens, compared to Group 1 (*Der p 1* and *Der f 1*), is consistent with existing literature and is primarily explained by the higher amino acid sequence identity and greater IgE cross-reactivity observed between Group 2 components (26). In high-exposure regions like the Mediterranean coast, this high homology means that the IgE response to Group 2 allergens is virtually indistinguishable between DP and DF.

While this study offers valuable insights into the immunological mechanisms underlying allergic diseases, it is important to note that multivariate regression models demonstrated a weak model fit and limited predictive capacity. Consequently, these models should be interpreted as exploratory rather than definitive predictive tools. Nevertheless, several noteworthy associations emerged from the analysis. Existing literature presents conflicting evidence regarding the relationship between age and HDM sensitization, with some studies reporting no significant age-related differences in sIgE responses (16, 23), while others have identified clear associations (13).

Our findings suggest that, at least within the studied cohort, sensitization profiles may exhibit changes over time. Specifically, *Der f 1* sensitization was significantly associated with younger individuals, and profile 2—characterized by multi-allergen reactivity—showed a strong correlation with younger age (<30 years) (Table 6).

**Table 6:** Summary of multivariate analysis of sIgE components with demographics and clinical characteristics.

	Der p 1	Der f 1	Der f 2	Der p 7	Der p 10	Der p 23
Age	Lower probability of positive result	Lower probability of positive result	Lower probability of positive result	Lower probability of positive result	Lower probability of positive result	Lower probability of positive result
Concomitant medication	Higher probability of positive result	Higher probability of positive result				
Sex (female)				Lower probability of positive result	Lower probability of positive result	
Asthma	Higher probability of positive result		Higher probability of positive result			Higher probability of positive result
Rhinitis intensity	Higher probability of positive result	Higher probability of positive result				Higher probability of positive result
Rhinitis control					Lower probability of positive result	

Recent evidence in pediatric populations has underscored how mite allergen recognition evolves with age and exposure duration (27, 28). However, it is important to note that our findings may not be directly comparable to purely pediatric cohorts given our predominantly adult population (median age 29). These differences reinforce the hypothesis that while the breadth of sensitization may increase during childhood, these patterns might stabilize or follow different dynamics as patients reach adulthood in high-exposure Mediterranean regions.

Results indicated that there is no progressive increase in IgE, suggesting that specific IgE levels tend to remain stable in older individuals. Longitudinal monitoring of sIgE responses at multiple time points may be necessary to fully elucidate these dynamic changes.

Sex also appeared to influence sensitization patterns. Notably, *Der p 5* exhibited a strong positive association with female gender, whereas *Der p 23* was significantly less common in females. Although none of the predefined sensitization profiles reached statistical significance in sex-stratified analyses, these sex-specific differences remained consistent across multiple models, including those adjusted for rhinitis and asthma subgroups. These findings align with prior research documenting sex-based variations in HDM sensitization (13, 29) and highlight the need to consider sex-specific patterns in the clinical management of allergic diseases.

It is important to acknowledge several limitations when interpreting the findings of the study. First, the cross-sectional nature of the design prevents definitive conclusions about causality or the timing of observed relationships. Furthermore, the study focused solely on participants from the Valencian region, which may limit the generalization of the results to other populations or locations (due to biological, ethnic or environmental differences).

The exclusion of patients with clinically relevant sensitisations to allergens other than HDM may limit the generalisability of the findings to the broader, symptomatic polysensitised population. However, this approach was preferred to preventing misclassification bias and to isolating the clinical impact of *Dermatophagoides* components and avoiding confounding effects from other seasonal or perennial allergens on clinical variables like rhinitis control. Despite the above, our study population still reflects a degree of molecular complexity, with a high prevalence of IgE-positivity to pollens (68%) and other mites like *Lepidoglyphus destructor* (22%), even if these were not the primary drivers of clinical disease in this cohort.

Furthermore, although all participants resided in the Valencian Community (a region with climatic conditions highly conducive to HDM proliferation), residency in such an area does not necessarily equate to sustained, high-level exposure throughout a patient's entire lifespan. Some studies have reported that discrepancies between the environmental exposome and sensitisation profiles can be explained by variations in the duration of exposure and residential history (30).

This study is based on the ALEX^2^ multiplex assay. The use of different diagnostic platforms, such as ALEX^2^ vs. others like the ImmunoCAP ISAC112 can lead to variations in sIgE binding frequencies (31). Therefore, such methodological differences should be considered when comparing sensitization results across studies.

In terms of correlation analysis and the determination of sensitisation profiles, a correlation matrix provides a general overview. However, this is insufficient to fully define these complex sensitisation links. A hierarchical clustering analysis based on positivity to the various components would provide biostatistical robustness. However, the current sample size and the low prevalence of sensitisation to certain minor allergens, some of which have well-known pan-allergens that frequently represent cross-reactivity, would limit its interpretation. Therefore, it has been decided to exclude this from the analysis in the present study.

Finally, the sample size of this study and the multiple comparisons across different sensitization profiles and clinical outcomes may lead to an inflated Type I error rate. Consequently, the associations identified in our multivariate models should be interpreted as exploratory rather than as inferential conclusions.

Despite these constraints, this work fills an important gap in existing research, given the limited data on allergen sensitization in Southern Europe—especially in Mediterranean areas of Spain. These results could be used to create a preliminary map of the molecular complexity in high-exposure regions. However, this map would need to be validated in larger, adequately powered longitudinal cohorts.

## Conclusion

Overall, the findings of this study support the existence of multiple, overlapping sensitization pathways among patients allergic to *Dermatophagoides*, rather than a single dominant profile. This variability has direct implications for the design of personalized diagnostic and therapeutic strategies, including the selection of allergen components for immunotherapy. Recognizing the diversity of sIgE response patterns is essential for optimizing patient management and improving clinical outcomes in house dust mite respiratory allergy.

## Data Availability

The raw data supporting the conclusions of this article will be made available by the authors, without undue reservation.
